# High-resolution 3T magnetic resonance imaging and histological analysis of capsuloligamentous complex of the first metatarsophalangeal joint

**DOI:** 10.1186/s13018-021-02795-7

**Published:** 2021-10-24

**Authors:** Jin-e Wang, Rong-jie Bai, Hui-li Zhan, Wen-ting Li, Zhan-hua Qian, Nai-li Wang, Yuming Yin

**Affiliations:** 1 Department of Radiology, Peking University Fourth School of Clinical Medicine, Beijing, 100035 China; 2 Department of Radiology, Beijing Jishuitan Hospital, Beijing, 100035 China; 3 Institute of Basic Medical Sciences, Chinese Academy of Medical Sciences, School of Basic Medicine, Peking Union Medical College, Beijing, 100005 China; 4 Direct Radiology, 3501 Ocean Drive, Corpus Christi, TX 78411 USA

**Keywords:** Anatomy, Magnetic resonance imaging, Histological analysis, Capsuloligamentous complex, First metatarsophalangeal joint, Foot

## Abstract

**Background:**

There are discrepancies in the understanding of the structure of the capsuloligamentous complex of the first metatarsophalangeal joint (MTPJ); this study aims to investigate the differences with previous anatomical reports of high-resolution 3T magnetic resonance imaging (MRI) and histological analysis in illustrating the structure of the capsuloligamentous complex of the first MTPJ.

**Methods:**

Nine fresh frozen cadaveric feet specimens (from two women and three men; aged 32 to 58 years) were used in this study. All specimens underwent MR examination with T1-weighted imaging and T2-weighted spectral attenuated inversion recovery in three planes. Subsequently, all cadaveric feet specimens were sliced into 2-mm-thick sections. The MRI features of the capsuloligamentous complex of the first MTPJ were analyzed in these specimens. Hematoxylin–eosin and Masson’s trichrome staining methods were used to explore the histologic features of the capsuloligamentous complex of the first MTPJ.

**Results:**

Different from most previous studies, our results showed that the plantar plate could be divided into four portions including the central portion of the plantar plate, the intersesamoid, the sesamoid phalangeal and the metatarsosesamoid ligaments. The normal central portion of the plantar plate could be clearly visualized in the sagittal and coronal plane MR images. The intersesamoid ligament is a continuation of the central portion of the plantar plate on the sagittal plane on the gross specimen, the MR imaging, and the histological examination. On the coronal plane of the gross specimen and MR imaging, the sesamoid phalangeal ligaments and the central portion of the plantar plate can be seen as separate ligaments, but they appeared interwoven with the same continuous collagenous fibers on the histological analysis.

**Conclusion:**

High-resolution 3T MRI allows accurate demonstration of the different anatomical details of the capsuloligamentous complex of the first MTPJ from previous anatomical reports. The histological analysis provides further understanding of the structures of the capsuloligamentous complex of the first MTPJ from previous studies.

**Supplementary Information:**

The online version contains supplementary material available at 10.1186/s13018-021-02795-7.

## Background

The capsuloligamentous complex of the first MTPJ is composed of joint capsule, multiple ligaments, plantar plate, and supporting structures [1–3]. It is the main stabilizer of the first MTPJ, and injuries of the capsuloligamentous complex of the first MTPJ may lead to “turf toe” [4]. However, there are discrepancies in the understanding of the structure of the capsuloligamentous complex of the first MTPJ, which make the accurate diagnosis of turf toe a challenge. Unfamiliar with the complexity of the normal anatomy and anatomic variation may lead to failure in recognizing the injury. If a delayed diagnosis and treatment occur, subsequent prolonged recovery may result in the instability of the joint and may lead to significant functional disability [5–9].

Previously, magnetic resonance imaging (MRI) has been shown to be valuable for the visualization of the fine anatomic structures of the capsuloligamentous complex of the first MTPJ and evaluation of tendons, ligaments, and cartilaginous injuries related to turf toe [2, 10–15]. In this study, using human fresh frozen cadaveric feet specimens and correlating with the high-resolution 3T MR images and histological evaluation in the visualization and analysis of the anatomy of the capsuloligamentous complex of the first MTPJ, we found that the components of the plantar plate, the relations between the intersesamoid ligament and the central portion of the plantar plate and the relations between the sesamoid phalangeal ligaments and the central portion of the plantar plate were different with previous reports.

## Methods

The study was conducted in accordance with the *Declaration of Helsinki* and was approved by the Ethics Committee of the Institutional Review Board of Beijing Jishuitan Hospital (201703-10). The authors certify that they have obtained all appropriate consent forms, which were signed by the family members of the donor-cadavers.

### Cadaveric feet specimens

Nine fresh frozen cadaveric feet specimens were obtained from five cadavers (two women and three men; aged from 32 to 58 years at death, mean age 42 years) provided by the Institute of Basic Sciences, Chinese Academy of Medical Sciences. All specimens included the whole ankle joint and foot and were kept at − 42 °C (Haier BioMedical, DW-40W100, Qingdao, China). All specimens were allowed to thaw for 24 h at room temperature before the MRI analysis. A anteroposterior and lateral X-ray analysis of nine feet specimens was performed to exclude the presence of articular disorders or osseous abnormalities.

### Magnetic resonance imaging

MR was performed with a 3T MRI Unit (5680 DA Best, Philips Medical Systems, Netherlands). A 16-channel head-and-neck receiver-only coil was used, centered on the forefoot by the laser mark. The MRI examination includes the following sequences: T1-weighted image (T1WI) and T2-weighted spectral attenuated inversion recovery (T2-SPAIR) in the coronal, transverse, and sagittal planes. In order to evaluate the detail of the capsuloligamentous complex of the first MTPJ, two specimens had additional sequences with smaller field of view (FOV: 6 cm × 6 cm) including T1WI and proton density-weighted imaging with fat suppression (PD-FS) in sagittal and coronal planes. Detailed parameters are listed in Additional file 1: Table S1.

### Anatomic specimen preparation

All cadaveric feet were frozen for at least 24 h at − 42 °C after obtaining the MR imaging. Subsequently, specimens were sliced into 2-mm-thick sections with a stainless-steel band saw (American Meat Equipment Corp, Montebello, USA); the section thickness is the same as the MR images. Nine specimens were sectioned as follows: sagittal (*n* = 3), transverse (*n* = 3), and coronal (*n* = 3) planes. Photographs of each section of the specimens were obtained.

### Histological examination

The histologic slice was fixed in 10% neutral phosphate-buffered formalin (NBF), dehydrated in ascending ethanol, decalcified with ethylenediaminetetraacetic acid (EDTA) and formic acid, and embedded into a paraffin wax block for histological examination. The 4-µm sequential transverse sections were stained with hematoxylin and eosin (HE) and Masson’s trichrome at original magnifications of 4× and 200× and then observed under the light microscope.

### Magnetic resonance imaging and light microscopic observation: anatomic comparison and analysis

All the MR images were interpreted by two musculoskeletal radiologists with 10–15 years of experience. The features of the capsuloligamentous complex of the first MTPJ on MRI were compared with those derived from examination of the anatomic segments of the cadaveric specimens.

Identification of each structure of the capsuloligamentous complex of the first MTPJ on MRI was by confirming the linear or curved hypointense signal structure in the expected location on MR imaging. Diagrams were drawn according to the appearance of the corresponding MR images.

Two musculoskeletal radiologists measured the capsuloligamentous complex of the first MTPJ of the MR images independently. All measurements were taken three times in the middle of their respective courses of the optimal MRI images plane [1, 16].

The data are presented as mean ± standard deviation. Interobserver agreement regarding measurements was assessed using the intraclass correlation coefficient (ICC). ICC value less than 0.20 was rated as slight agreement, between 0.21 and 0.40 as fair agreement, between 0.41 and 0.60 as moderate agreement, between 0.61 and 0.80 as good agreement, and between 0.81 and 1.00 as very good interobserver agreement.

The structure of the capsuloligamentous complex of the first MTPJ was identified by confirming the collagen fibers on histological examination including the central portion of the plantar plate, the intersesamoid, the sesamoid phalangeal, the accessory sesamoid ligaments, and abductor and adductor hallucis tendons.

## Results

### Anatomy of the capsuloligamentous complex of the first MTPJ

Analysis of the gross specimens confirmed that capsuloligamentous complex of the first MTPJ was composed of plantar plate, collateral ligaments, and supporting structures (Figs. 1–8).

**Fig. 1:**
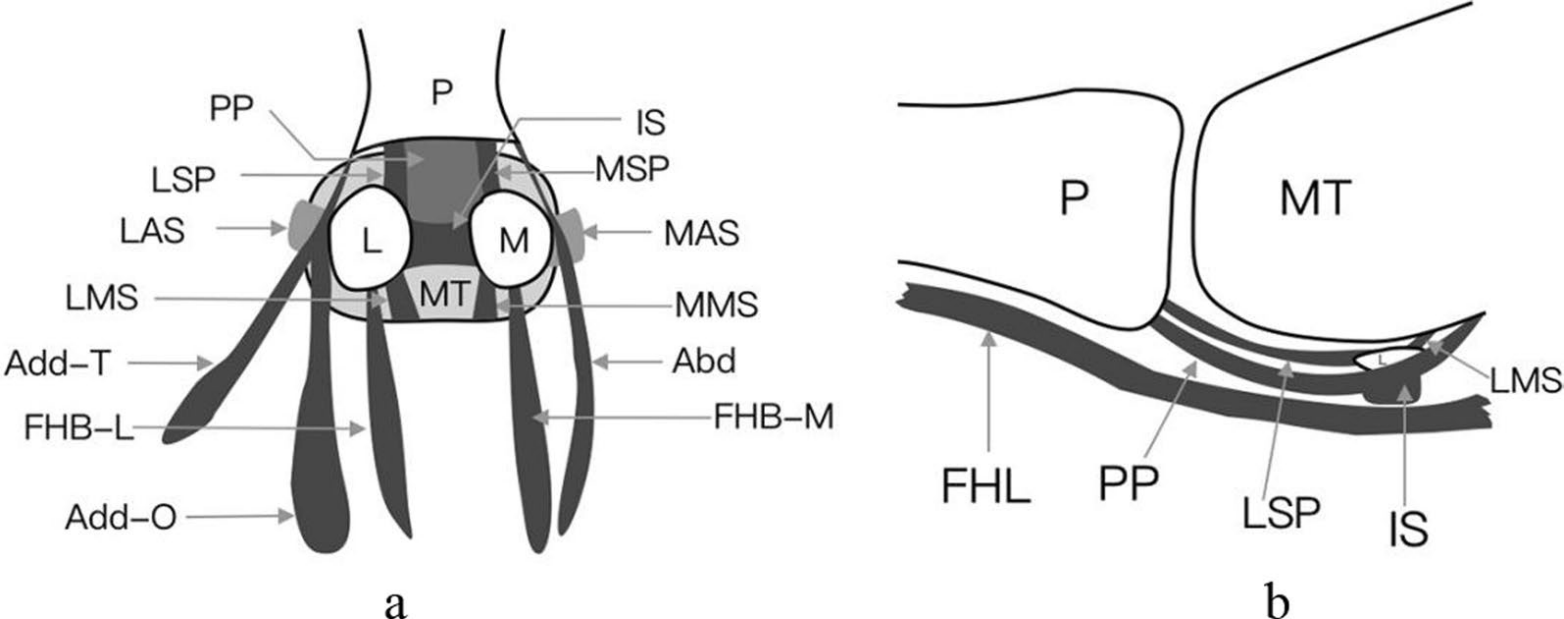
Schematic diagram of capsuloligamentous complex of the first MTPJ in the foot. **a** Anatomy of the first MTPJ, dorsal view; **b** Anatomy of the first MTPJ, medial side of the proximal midsagittal images. (Abd = abductor hallucis tendon; Add-O = oblique head of adductor hallucis tendon; Add-T = transverse head of adductor hallucis tendon; FHB-M = medial head of flexor hallucis brevis tendon; FHB-L = lateral head of the flexor hallucis brevis tendon; FHL = flexor hallucis longus tendon; IS = intersesamoid ligament; L = lateral sesamoid; LAS = lateral accessory sesamoid ligament; LMS = lateral metatarsosesamoid ligament; LSP = lateral sesamoid phalangeal ligament; M = medial sesamoid; MAS = medial accessory sesamoid ligament; MMS = medial metatarsosesamoid ligament; MSP = medial sesamoid phalangeal ligament; MT = metatarsal; P = phalanx; PP = central portion of the plantar plate)

#### The plantar plate of the capsuloligamentous complex of the first MTPJ

The plantar plate could be divided into four portions (central portion of the plantar plate, intersesamoid, sesamoid phalangeal and metatarsosesamoid ligaments) by the embedded sesamoids (Fig. 1).

**Fig. 2:**
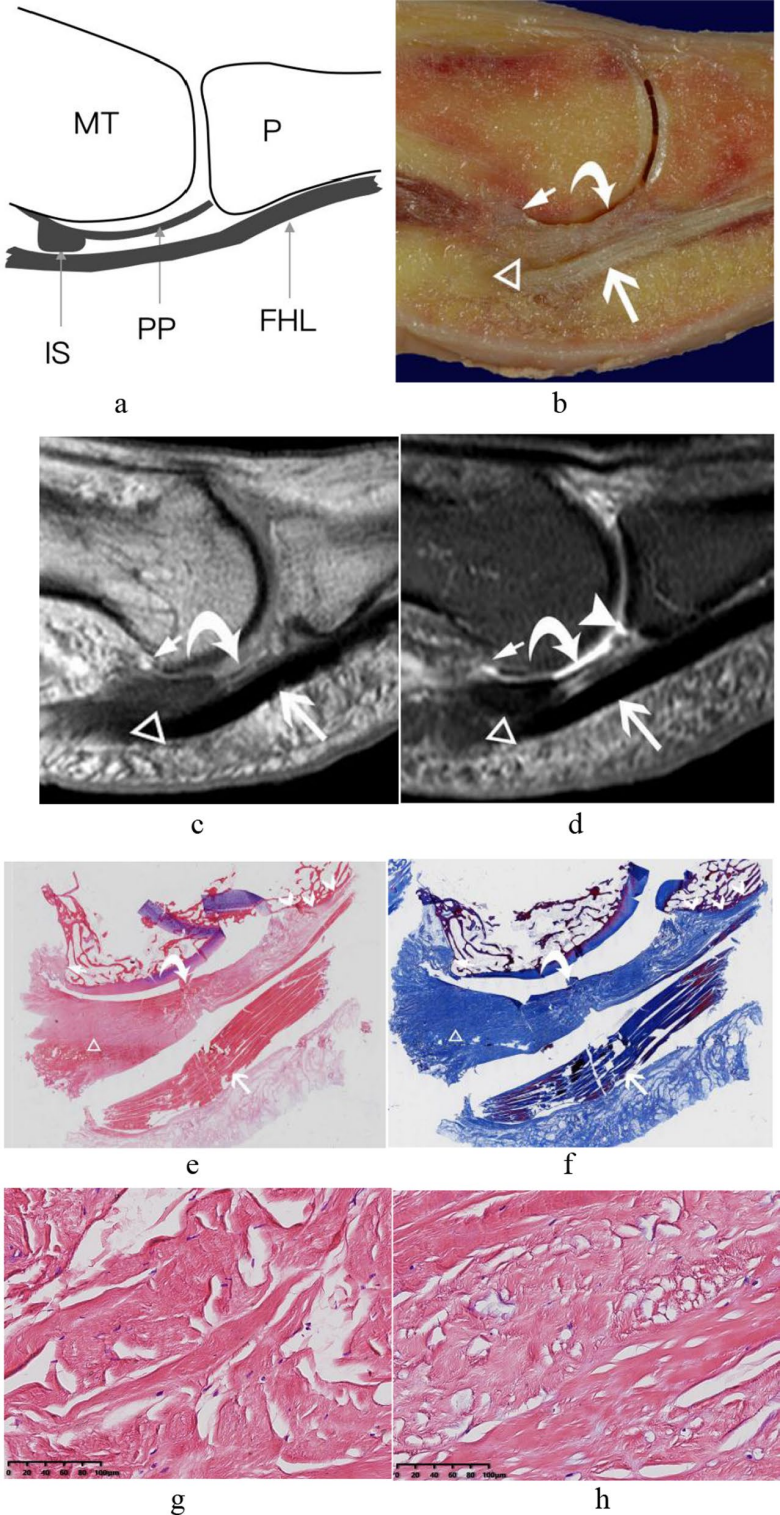
The central portion of the plantar plate and intersesamoid ligament of the first MTPJ from a left foot specimen (33 years old). **a** Midsagittal schematic diagram of first MTPJ; **b** sagittal anatomic slice; **c** sagittal T1WI image of the MTPJ; **d** sagittal PD-FS image of the MTPJ. White curved arrow: The central portion of the plantar plate coursed from metatarsal neck (white short arrow) to the proximal phalangeal base. Note a normal, smooth, well-defined distal recess (arrowhead) at the insertion of the proximal phalangeal base of the central portion of the plantar plate. (The white long arrow = flexor hallucis longus tendon; White triangle = The intersesamoid ligament). **e** and **f** HE and Masson’s trichrome staining of the first MTPJ (original magnification ×4). The white short arrow and arrowhead: the proximal and distal attachment of the central portion of the plantar plate. **g** and **h** HE staining of the central portion of plantar plate and the intersesamoid ligament; They appeared interwoven with the same continuous collagenous fibers that with a small number of chondroid metaplasia (HE, original magnification × 200). (FHL = flexor hallucis longus tendon; IS = intersesamoid ligament; MT = metatarsal; P = phalanx; PP = central portion of the plantar plate.)

**Fig. 3:**
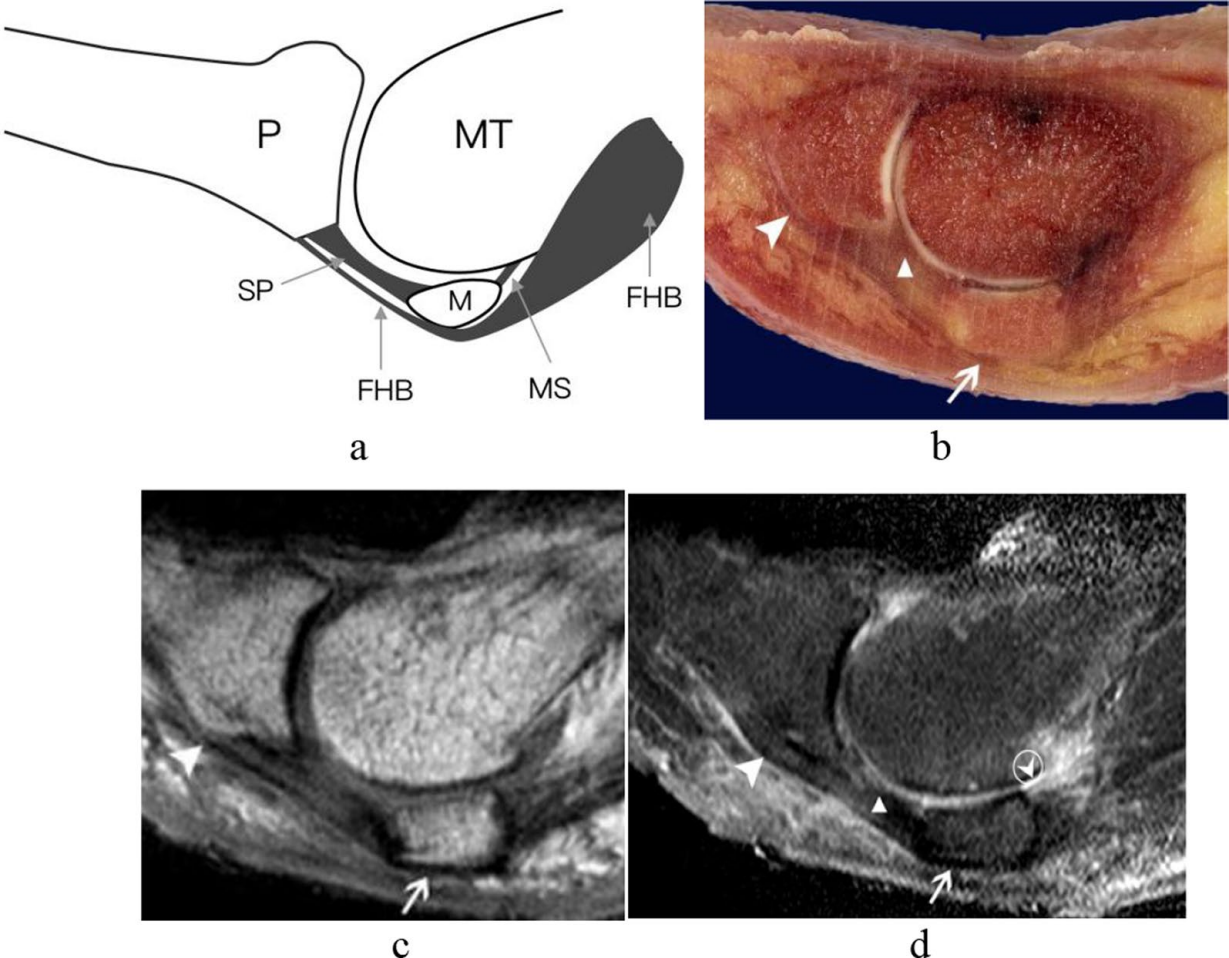
The medial sesamoid phalangeal ligament, metatarsosesamoid ligament, the tendon of medial head of the flexor hallucis brevis from a left foot specimen (58 years old). **a** Parasagittal schematic diagram through the medial sesamoid of first MTPJ; **b** sagittal anatomic slice; **c** and **d** sagittal T1WI and PD-FS image of the MTPJ. White triangle = The medial sesamoid phalangeal ligament; white arrowhead in circle = The medial metatarsosesamoid ligament; white arrow: The tendon of medial head of the flexor hallucis brevis inserted to the plantar margin of the medial sesamoid, and continued distally to attach to the proximal phalangeal base (white arrowhead). (FHB = the flexor hallucis brevis; M = medial sesamoid; MS = metatarsosesamoid ligament; MT = metatarsal; P = phalanx; SP = sesamoid phalangeal ligament)

**Fig. 4:**
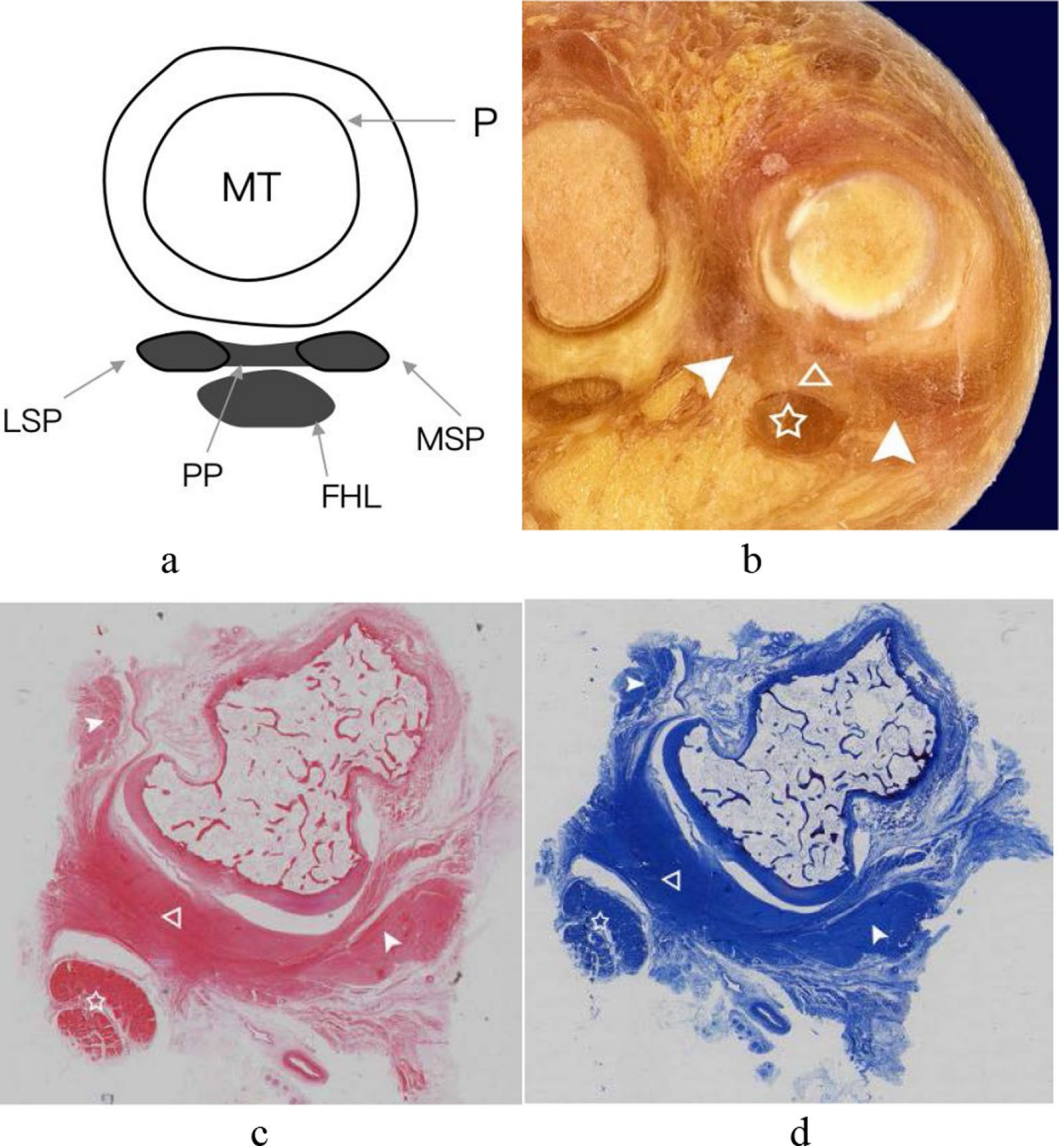
The central portion of the plantar plate and sesamoid phalangeal ligaments of the first MTPJ from a right foot specimen (32 years old). **a** Coronal schematic diagram of first MTPJ; **b** coronal anatomic specimen; the central portion of the plantar plate (white triangle) was separate fibrous band located between the medial and lateral sesamoid phalangeal ligaments (white arrowheads); **c** and **d** HE and Masson’s trichrome staining of the first MTPJ (original magnification × 4). The central portion of the plantar plate (white triangle) and the sesamoid phalangeal ligaments (white arrowheads) were composed of the same continuous collagen fibers and showed eosinophilic features by HE, blue with Masson’s trichrome staining, respectively. (White pentagram = the flexor hallucis longus tendon; FHL = flexor hallucis longus tendon; LSP = lateral sesamoid phalangeal ligament; MSP = medial sesamoid phalangeal ligament; MT = metatarsal; P = phalanx; PP = central portion of the plantar plate.)

**Fig. 5:**
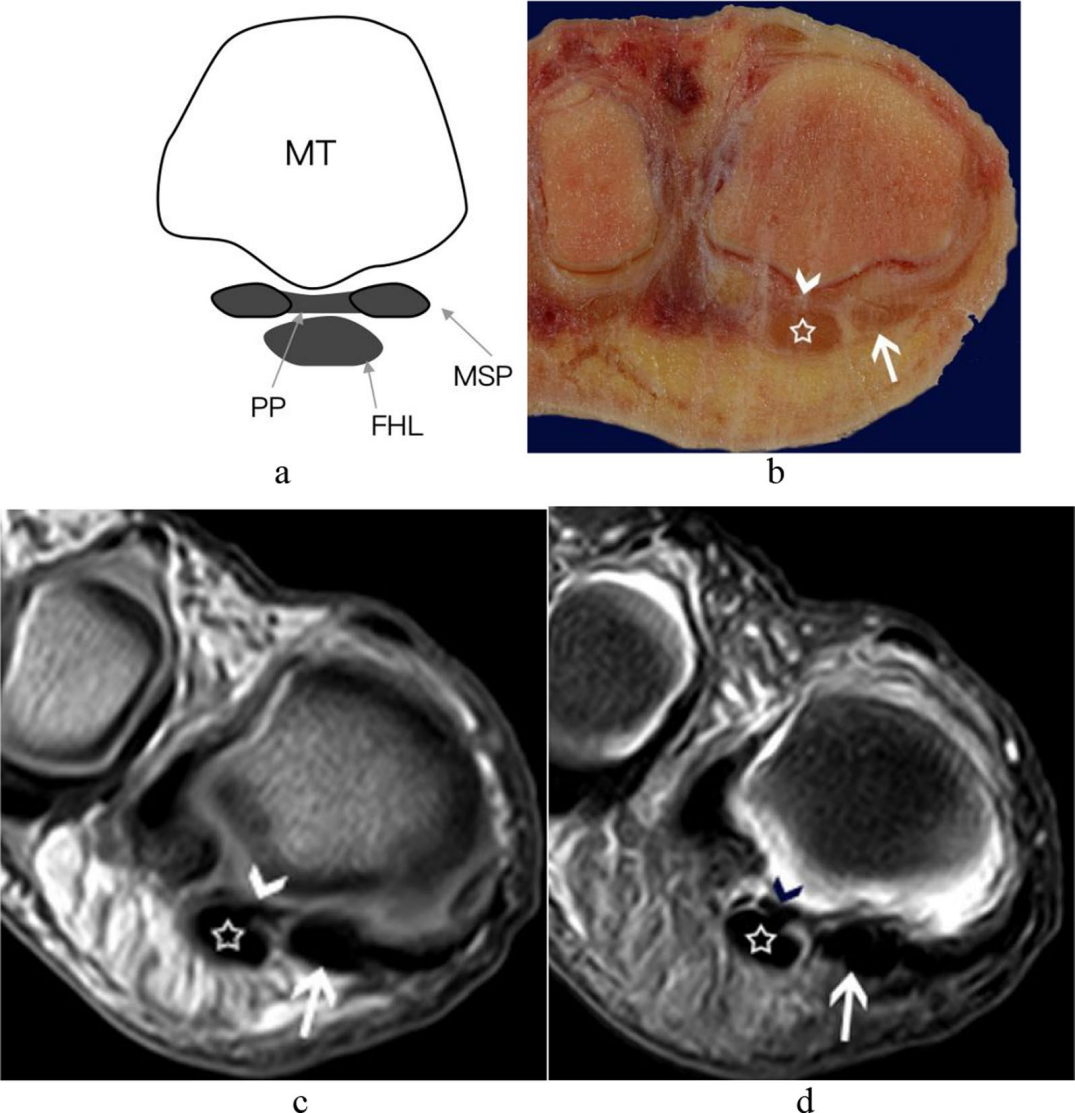
The central portion of the plantar plate and medial sesamoid phalangeal ligament of the first MTPJ from a right foot specimen (45 years old). **a** Coronal anatomic comparison slice; **b** coronal anatomic slice; **c** coronal T1WI image of the foot; **d** coronal T2-SPAIR image of the foot. The central portion of the plantar plate (arrowhead) was recognized as curved hypointense signal band on the dorsal of the flexor hallucis longus tendon (white pentagram). (The white arrow = the medial sesamoid phalangeal ligament; FHL = flexor hallucis longus tendon; MSP = medial sesamoid phalangeal ligament; MT = metatarsal; PP = central portion of the plantar plate)

The central portion of the plantar plate originated from the metatarsal neck and extended toward the plantar side of the proximal phalangeal base distally (Fig. 2). The posterior midline aspect of the plantar plate, locating between the medial and lateral sesamoids, was referred to as the intersesamoid ligament. At the periphery of the plantar plate, it was divided into proximal metatarsosesamoid ligament and distal sesamoid phalangeal ligament with the intervening sesamoids: the former one extended from the metatarsal neck and inserted on the proximal margin of sesamoid, the latter one originated from the distal aspect of the sesamoid to the proximal phalangeal base (Fig. 3). The central portion of the plantar plate was a separate fibrous band located between the medial and lateral sesamoid phalangeal ligaments in the coronal plane (Figs. 4 and 5), but it continued with the intersesamoid ligament in the sagittal plane (Fig. 2).

#### The collateral ligaments of the capsuloligamentous complex of the first MTPJ

**Fig. 6:**
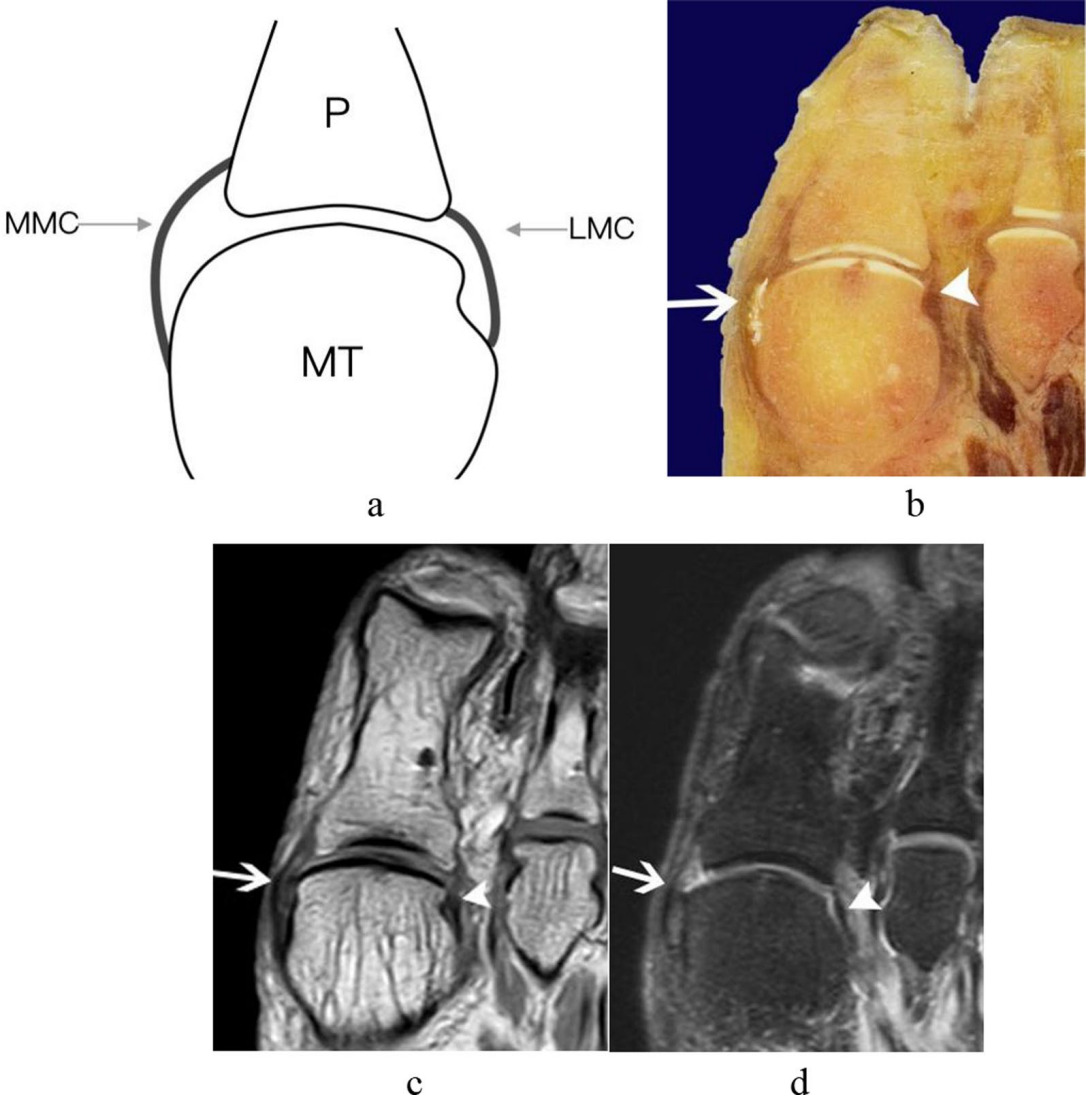
The medial and lateral main collateral ligaments from a right foot specimen (33 years old). **a** Transverse schematic diagram of first MTPJ; **b** transverse anatomic slice; **c** transverse T1WI image of the foot; **d** transverse T2-SPAIR image of the foot. The medial (white arrow) and lateral main collateral ligaments (white arrowhead) were recognized as curved hypointense signal band coursing from the metatarsal head to the proximal phalangeal base. (LMC = lateral main collateral ligament; MMC = medial main collateral ligament; MT = metatarsal; P = phalanx.)

**Fig. 7:**
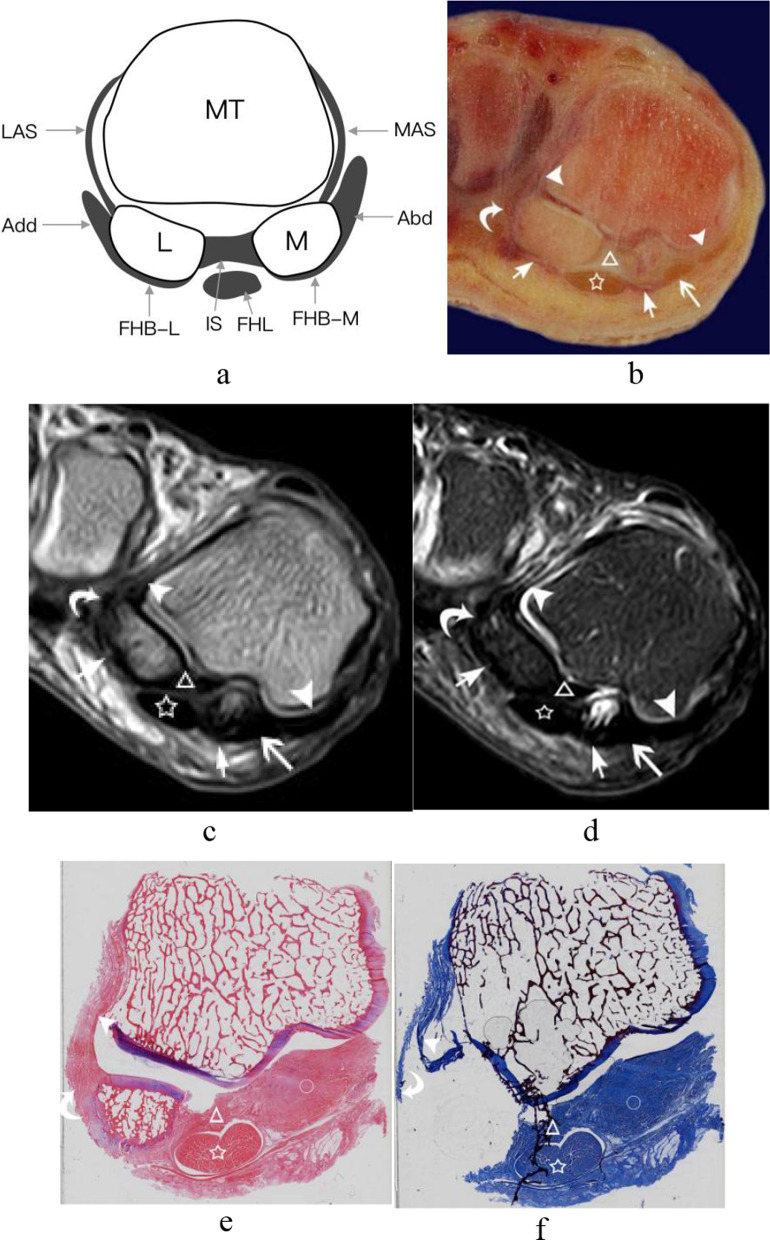
The capsuloligamentous complex of the first MTPJ from a right foot specimen (45 years old). **a** Coronal schematic diagram through the sesamoid bones of first MTPJ; **b** coronal anatomic slice; **c** and **d** coronal T1WI and T2-SPAIR image of the foot; **e** and **f** HE and Masson’s trichrome staining of the first MTPJ (original magnification × 4). White triangle = The intersesamoid ligament; white pentagram = flexor hallucis longus tendon; white arrowheads = the accessory sesamoid ligaments; white short arrows = the medial and lateral head of the flexor hallucis brevis tendons; white long arrow and white curved arrow = The tendons of abductor hallucis and adductor hallucis; white circle = The medial sesamoid phalangeal ligament. (Abd = abductor hallucis tendon; Add = adductor hallucis tendon; FHB-M and FHB-L = medial and lateral head of flexor hallucis brevis tendon; FHL = flexor hallucis longus tendon; IS = intersesamoid ligament; L = lateral sesamoid; LAS = lateral accessory sesamoid ligament; M = medial sesamoid; MAS = medial accessory sesamoid ligament; MT = metatarsal.)

The collateral ligaments consisted of two main collateral ligaments and two accessory sesamoid ligaments. The medial main collateral ligaments originated from the medial aspect of the metatarsal head to attach to the medial aspect of the proximal phalangeal base. The lateral main collateral ligaments originated from the lateral aspect of the metatarsal head to attach to the lateral aspect of the proximal phalangeal base (Fig. 6). The accessory sesamoid ligaments extended from the same proximal attachments with the main collateral ligaments to the periphery of the sesamoid bone (Fig. 7).

#### The supporting structures of the capsuloligamentous complex of the first MTPJ

**Fig. 8:**
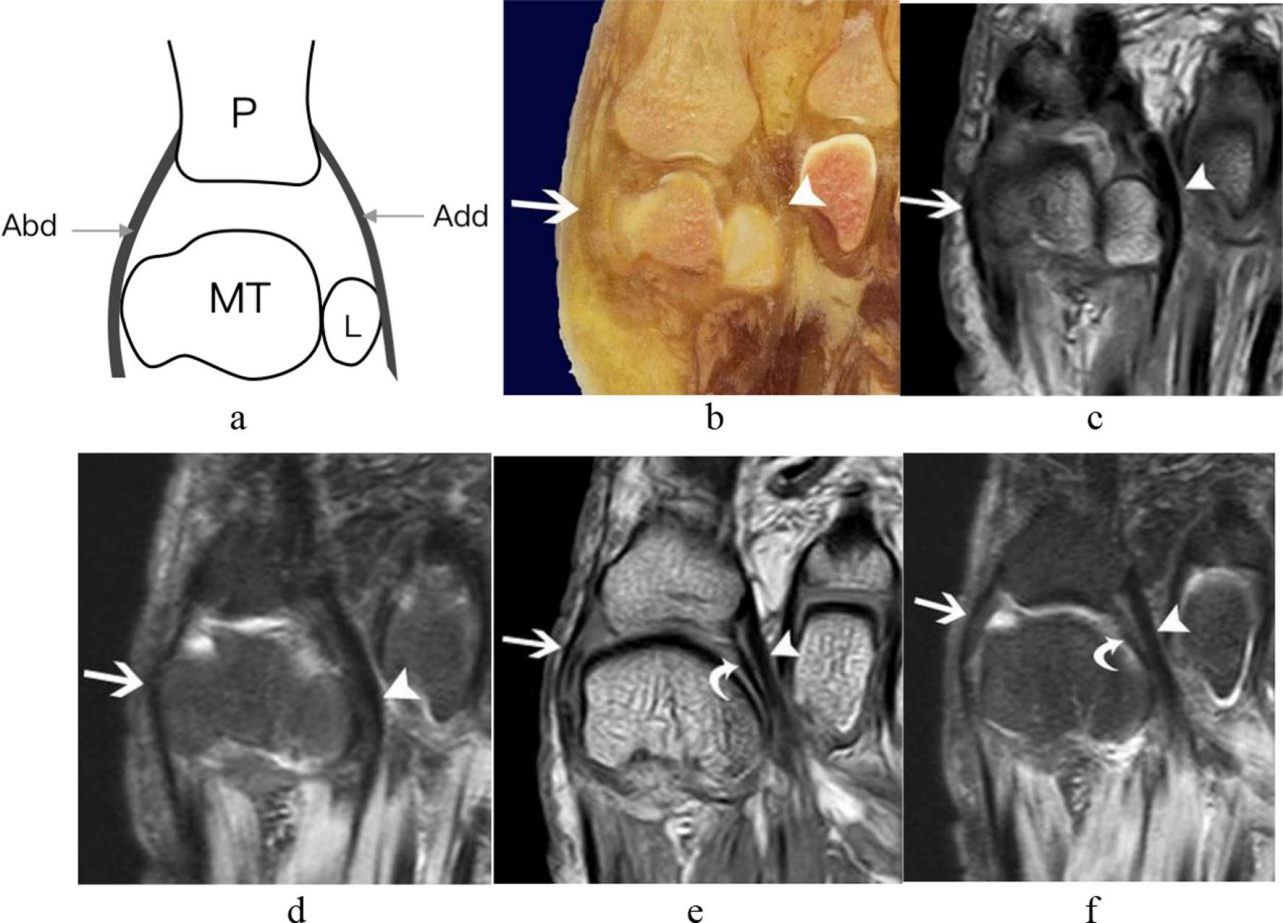
The tendons of the adductor hallucis and abductor hallucis tendon from a right foot specimen (33 years old). **a** Transverse schematic diagram of first MTPJ; **b** transverse anatomic slice; **c** and **e** transverse T1WI image of the foot; **d** and **f** transverse T2-SPAIR image of the foot. (White arrow shows the abductor hallucis tendon; white arrowhead shows the adductor hallucis tendon. The lateral main collateral ligament (white curved arrow) extended distally to the lateral base of the proximal phalanx parallel to tendon of adductor hallucis. (Abd = abductor hallucis tendon; Add = adductor hallucis tendon; L = lateral sesamoid; MT = metatarsal; P = phalanx)

The supporting structures of the capsuloligamentous complex of the first MTPJ were composed of the medial and lateral heads of the flexor hallucis brevis tendons and abductor and adductor hallucis tendons. The medial and lateral heads of the flexor hallucis brevis tendons attached to the plantar aspect of the medial and lateral sesamoids, respectively, and continued distally to attach to the proximal phalangeal base (Fig. 7). The abductor hallucis tendons attached to the medial side of the medial sesamoids, forming a conjoined tendon with the flexor hallucis brevis to insert into the plantar aspect of the medial side of the proximal phalangeal base. The adductor hallucis tendons attached to the lateral side of the lateral sesamoids, forming a conjoined tendon with the flexor hallucis brevis to insert into the plantar aspect of the lateral side of the proximal phalangeal base (Fig. 8).

### MRI investigation of the capsuloligamentous complex of the first MTPJ

On MR images, the normal structures of the capsuloligamentous complex of the first MTPJ showed hypointense signal in all the sequences including T1‑weighted, T2-SPAIR image, and PD-FS.

In the midsagittal plane, the central portion of the plantar plate continued with the intersesamoid ligament on the dorsal aspect of the flexor hallucis longus tendon. We can see a normal hyperintense signal recess at the insertion of the proximal phalangeal base of the central portion of the plantar plate in the midsagittal plane of all five cadaveric feet, which should not be mistaken as a tear (Fig. 2). In the parasagittal plane through the sesamoid bone, the sesamoid phalangeal ligament, metatarsosesamoid ligament, and the flexor hallucis brevis tendon were best demonstrated (Fig. 3).

In the coronal plane at the level of the sesamoid bones, the intersesamoid ligament, the accessory sesamoid ligaments, and the supporting structures were all clearly appreciated (Fig. 7). In the coronal plane at the level of the distal to the sesamoid bone, the central portion of the plantar plate manifested as a separate hypointense signal band located between the medial and the lateral sesamoid phalangeal ligaments (Fig. 5).

In the transverse plane at the level of the sesamoid bones, the intersesamoid ligament, the abductor and adductor hallucis tendons were all clearly appreciated (Fig. 8). In the transverse plane at the level of MTPJ, the medial and lateral main collateral ligaments were clearly observed; the lateral one extended distally to the lateral base of the proximal phalanx parallel to the tendon of adductor hallucis (Figs. 6 and 8).

The measurement results of the structures of the capsuloligamentous complex of the first MTPJ are listed in Table 1.

**Table 1:** Measurement results of the capsuloligamentous complex of the first MTPJ

	No.	Mean (mm)	SD (mm)	Range (mm)
Central portion of the plantar plate				
Length	6	19.6	2.4	16.4–22.0
Width	7	2.9	1.3	1.4–4.8
Depth	8	0.5	0.2	0.3–1.1
Intersesamoid ligament				
Width	9	3.4	0.7	2.2–4.2
Depth	9	2.5	0.6	1.4–3.4
Medial sesamoid phalangeal ligament;				
Length	9	11.0	2.1	7.5–14.3
Width	9	3.4	0.8	2.3–4.5
Depth	9	3.2	0.6	2.6–4.2
Lateral sesamoid phalangeal ligament;				
Length	9	7.9	0.7	6.7–8.8
Width	9	3.7	0.7	2.8–5.2
Depth	9	2.8	0.8	1.1–3.6
Medial metatarsosesamoid ligament;				
Length	5	5.4	0.9	3.9–6.3
Width	5	0.3	0.1	0.2–0.4
Lateral metatarsosesamoid ligament;				
Length	5	6.1	1.9	3.6–7.9
Width	5	0.3	0.0	0.3–0.4
Medial main collateral ligament				
Length	9	14.5	2.0	10.1–16.2
Width	9	2.0	0.5	1.2–2.7
Lateral main collateral ligament				
Length	7	12.3	2.2	9.7–14.7
Width	7	1.7	0.4	1.3–2.4
Medial head of the flexor hallucis brevis tendon				
Width	3	2.5	0.7	1.9–3.3
Depth	3	0.4	0.2	0.3–0.7
Lateral head of the flexor hallucis brevis tendon				
Width	2	2.9	0.4	2.6–3.1
Depth	2	0.3	0.1	0.3–0.4
Adductor hallucis tendon				
Width	5	5.7	1.6	3.4–7.4
Abductor hallucis tendon				
Width	5	2.8	0.3	2.4–3.1

*Abbreviations*: No, number of the structures; SD, standard deviation

Interobserver agreement ICC values were used to assess interobserver agreement between reader 1 and reader 2 with regard to quantitative measurements. ICC values ranged from 0.46 for the length of the central portion of the plantar plate to 0.97 for the depth of the intersesamoid ligament, rated as interobserver agreement from moderate to very good. Most ICC values were rated as good or very good.

### Histological study of the capsuloligamentous complex of the first MTPJ

The histological examination showed that the central portion of the plantar plate, intersesamoid, sesamoid phalangeal and accessory sesamoid ligaments, the tendons of abductor and adductor hallucis of the capsuloligamentous complex of the first MTPJ were all composed of compact collagen fibers. In HE results, the collagen fibers showed eosinophilic features, appeared red in color. The collagen fibers appeared blue with Masson’s trichrome staining (Figs. 2, 4 and 7). The central portion of the plantar plate and intersesamoid ligament were composed of the same continuous fiber on the midsagittal slice (Fig. 2e). The central portion of the plantar plate and sesamoid phalangeal ligaments were composed of the same continuous fiber on the coronal slice (Fig. 4). At high magnification (×200), the central portion of the plantar plate, intersesamoid and sesamoid phalangeal ligaments appeared interwoven with the same continuous transverse and longitudinal bundles of collagenous fibers with a small number of chondroid metaplasia (Fig. 2). The central portion of the plantar plate, intersesamoid, sesamoid phalangeal and accessory sesamoid ligaments, and the tendons of adductor and abductor hallucis of the capsuloligamentous complex of the first MTPJ showed hypointense signal on all MR images except the normal distal recess of the central portion of the plantar plate that showed hyperintense signal.

## Discussion

The capsuloligamentous complex of the first MTPJ plays an important role in stabilizing the first MTPJ. The complex anatomy of the capsuloligamentous complex of the first MTPJ makes the accurate imaging diagnosis of turf toe very challenge. MRI plays a crucial role in assessing the anatomic details of capsuloligamentous complex of the first MTPJ and has become the most critical imaging modality for visualization of tendons, ligaments, and cartilaginous injuries related to turf toe [1, 2, 10–18]. Our histological analysis of the first MTPJ provides a different understanding of the MRI features from previous studies and clarifies the discrepancy.

Previous reports [19, 20] showed that the plantar plate of the first MTPJ differed from that of the lesser ones (2–5) in that it spanned between the proximal phalangeal base and metatarsal head with the intervening hallux sesamoids. There are discrepancies when describing the components of the plantar plate [11, 13, 16, 21–25]. Some studies [2, 15] reported that the plantar plate arose from the distal margin of the intersesamoid ligament and extended distally toward the base of the proximal phalanx. They believed that the plantar plate could not be visualized clearly unless injury caused edema or thickening around it. In our opinion, the plantar plate can be divided into four portions including the central portion of the plantar plate, the intersesamoid, sesamoid phalangeal and metatarsosesamoid ligaments. The central portion of the plantar plate can be clearly visualized in the sagittal and coronal planes in normal cadaver. Other study [1] reported that there was a normal distal recess at the proximal phalangeal insertion of the plantar plate. In our study, we could see the hyperintense signal recess in five cadaveric feet on midsagittal MR images without arthrography, as described in the previous report [11]. This normal appearance should not be recognized as a tear or degeneration. This recess is a smooth, well-defined hyperintense signal region at the proximal phalangeal insertion of the central portion of the plantar plate. The degeneration or tear of the plantar plate usually shows increased signal intensity within the plantar plate, but it is usually poorly defined and irregular.

There are discrepancies when describing the relations between the intersesamoid ligament and the plantar plate in the literature. Allen et al. [6] described that the intersesamoid ligament was on the plantar aspect of the plantar plate. However, the more popular illustration was that the intersesamoid ligament was on the dorsal aspect of the plantar plate [26, 27]. Sanders et al. [28] described that the intersesamoid ligament was the central part of the plantar plate. Tolu et al. [29] illustrated that the intersesamoid ligament was a proximal thickening of the plantar plate. Srinivasan et al. [11] considered that the intersesamoid ligament and the plantar plate were made up of the same contiguous fibrocartilage. In our study, we found that the intersesamoid ligament continued with the central portion of the plantar plate on the sagittal plane of the gross specimen and MR imaging. The central portion of the plantar plate and intersesamoid ligament were composed of the same continuous fiber on the histological slice and appeared interwoven with the same continuous collagenous fibers at high magnification. The previous study reported that the thickness of the intersesamoid ligament was 3.1 ± 0.5 mm [16], which was similar to our result (2.5 ± 0.6 mm).

In our study, the sesamoid phalangeal ligaments and the central portion of the plantar plate were separate ligaments on the coronal plane of the gross specimen and MR imaging, but they appeared interwoven with the same continuous collagenous fibers on the histological slice. Srinivasan et al. [11] also stated that the sesamoid phalangeal and intersesamoid ligaments were not separate ligaments; they were made up of the same continuous fibrocartilage as the plantar plate. Previous researchers [2] described that sesamoid phalangeal ligaments were generally much wider than the metatarsosesamoid ligaments. We found that the length, width, and depth of sesamoid phalangeal ligaments were all greater than those of the metatarsosesamoid ligaments in our study.

Linklater et al. [21] suggested that collateral ligaments were composed of sesamoid collateral ligament and collateral ligament proper. However, Srinivasan et al. [11] had the same view with us that the collateral ligaments consisted of the main collateral ligaments and the accessory sesamoid ligaments. In our study, the mean width of the medial was (2.0 ± 0.5 mm), larger than the mean width of the lateral main collateral ligament (1.7 ± 0.4 mm). Theumann et al. [1] have the similar result with ours, except that both medial and lateral main collateral ligaments are thicker than ours. In their result, the lateral main collateral ligament (2.1 ± 0.2 mm) was thinner than the medial one (3.1 ± 0.4 mm). This discrepancy may be due to the ethnic differences of the studied subjects.

So far, few studies have concentrated on the histological feature of the capsuloligamentous complex of the first MTPJ. Brenner et al. [30] illustrated that the intersesamoid ligament was mainly composed of transverse bundles of collagenous fibers with little vertical and longitudinal fiber bundles; the insertion bundles to the sesamoids of the intersesamoid ligament were crisscrossing. Gregg et al. [31] stated that the lesser (2–5) metatarsophalangeal joint plantar plate collagen bundles appeared interwoven with longitudinal fiber bundles. In our study, the central portion of the plantar plate, intersesamoid and sesamoid phalangeal ligaments appeared interwoven with the same continuous transverse and longitudinal bundles of collagenous fibers. Previous reports [32–35] illustrated that normal tendons and ligaments had hypointense signal in all the conventional MRI sequences because the alignment of collagen molecules and water led to dipole interactions that shorten the T2 relaxation time to 1–2 ms and decayed the MR signal. In our study, the central portion of the plantar plate, intersesamoid, sesamoid phalangeal and accessory sesamoid ligaments, and the tendons of adductor and abductor hallucis of the capsuloligamentous complex of the first MTPJ were composed of collagen fibers; they all showed hypointense signal in MRI results except the normal distal recess of the central portion of the plantar plate.

There are some limitations in our study. First, the number of specimens included was relatively small. Second, the capsuloligamentous complex of the first MTPJ of the specimens could be placed in the center of the coil, which was helpful to get better images, but is not always possible in clinical settings. Third, the study did not involve live volunteers, which may provide more anatomical details of the capsuloligamentous complex of the first MTPJ in the real clinical setting.

## Conclusions

In conclusion, using the high-resolution 3T MRI combined with histological analysis, our study clearly illustrated the normal complex anatomy of the capsuloligamentous complex of the first MTPJ, which was different from previous studies. In our study, we found that the central portion of the plantar plate and intersesamoid ligament were composed of the same continuous fiber and the intersesamoid ligament continued with the central portion of the plantar plate and appeared interwoven with the same continuous collagenous fibers. All the structures can be clearly seen on MR images.

## Supplementary Information

**Additional file 1. Supplementary Table 1.** Parameters of standard high resolution 3T MRI Sequences for head-and-neck receiver-only coil (16-channel).

## Data Availability

All data generated or analyzed during this study are included in this published article [and its supplementary information files].

## Abbreviations

Abd: Abductor hallucis tendon
Add: Adductor hallucis tendon
Add-O: Oblique head of adductor hallucis tendon
Add-T: Transverse head of adductor hallucis tendon
EDTA: Ethylenediaminetetraacetic acid
FHB: The flexor hallucis brevis
FHB-M: Medial head of flexor hallucis brevis tendon
FHB-L: Lateral head of the flexor hallucis brevis tendon
FHL: Flexor hallucis longus tendon
HE: Hematoxylin and eosin
ICC: Intraclass correlation coefficient
IS: Intersesamoid ligament
L: Lateral sesamoid
LAS: Lateral accessory sesamoid ligament
LMC: Lateral main collateral ligament
LMS: Lateral metatarsosesamoid ligament
LSP: Lateral sesamoid phalangeal ligament
M: Medial sesamoid
MAS: Medial accessory sesamoid ligament
MMC: Medial main collateral ligament
MMS: Medial metatarsosesamoid ligament
MRI: Magnetic resonance imaging
MS: Metatarsosesamoid ligament
MSP: Medial sesamoid phalangeal ligament
MT: Metatarsal
MTPJ: Metatarsophalangeal joint
NBF: Neutral phosphate-buffered formalin
NSA: Number of signals acquired
P: Phalanx
PD-FS: Proton density-weighted imaging with fat suppression
PP: Central portion of the plantar plate
SP: Sesamoid phalangeal ligament
SPAIR: Spectral attenuated inversion recovery
T1WI: T1-weighted image
TE: Echo time
TR: Repetition time
