# Supplementary material for: High-resolution 3T magnetic resonance imaging and histological analysis of capsuloligamentous complex of the first metatarsophalangeal joint
Source: J Orthop Surg Res. 2021 Oct 24;16:638. doi: 10.1186/s13018-021-02795-7 (PMC8543817; doi:10.1186/s13018-021-02795-7)
Supplement: Supplementary file 1 — Additional file 1. Supplementary Table 1. Parameters of standard high resolution 3T MRI Sequences for head-and-neck receiver-only coil (16-channel). [file 13018_2021_2795_MOESM1_ESM.docx]

Supplementary Table 1. Parameters of standard high resolution 3T MRI Sequences for head-and-neck receiver-only coil (16-channel).

| Sequence | TR/TE (ms) | FOV (cm) | Matrix | NSA | Section thickness (mm) | Interslice space (mm) |
| --- | --- | --- | --- | --- | --- | --- |
| Foot Transverse T1WI | 642/30 | 10-15 | 428×340 | 2 | 2 | 0.2 |
| Foot Transverse T2-SPAIR | 7678/80 | 10-15 | 312×247 | 2 | 2 | 0.2 |
| Foot Sagittal T1WI | 668/30 | 6-15 | 428×340 | 2 | 2 | 0.2 |
| Foot Sagittal T2-SPAIR | 5071/80 | 7.5-15 | 312×247 | 2 | 2 | 0.2 |
| Foot Coronal T1WI | 668/30 | 10-15 | 428×340 | 2 | 2 | 0.2 |
| Foot Coronal T2-SPAIR | 9597/80 | 12.5-15 | 312×247 | 2 | 2 | 0.2 |
| MTPJ Sagittal T1WI | 5000/40-45 | 6 | 240×190 | 2 | 2 | 0.2 |
| MTPJ Coronal T1WI | 5000/40-45 | 6 | 240×190 | 2 | 2 | 0.2 |
| MTPJ Sagittal PD-FS | 5000/40-45 | 6 | 240×215 | 2 | 2 | 0.2 |
| MTPJ Coronal PD-FS | 5000/40-45 | 6 | 148×126 | 2 | 2 | 0.2 |

Abbreviations: SPAIR, spectral attenuated inversion recovery; PD-FS, Proton density-weighted imaging with fat suppression; TR, repetition time; TE, echo time; NSA, number of signals acquired
